# La ponction biopsie hépatique à Dakar: indications, complications et apport diagnostique - à propos de 70 cas

**DOI:** 10.11604/pamj.2014.17.85.2703

**Published:** 2014-02-03

**Authors:** Papa Souleymane Touré, Abdoulaye Léye, Madoky Maguette Diop, Mame Daouda Gueye, Yakham Mohamed Léye, Adama Berthé, Mamadou Mourtalla Ka

**Affiliations:** 1 Service de Médecine Interne Centre Hospitalier National de Pikine, Sis Ex Camp Militaire de Thiaroye, Dakar, Sénégal; 2 Université de Thiès, UFR des Sciences de la Santé, Ex lO éme RAIOM, Thiès, Sénégal

**Keywords:** Ponction-biopsie hépatique, echographie, apport diagnostique, Liver biopsy, echography, diagnostic contribution

## Abstract

**Introduction:**

Les objectifs de notre travail étaient de déterminer les indications, les complications et l'apport diagnostique de la ponction biopsie hépatique (PBH) transpariétale.

**Méthodes:**

Il s'agissait d'une étude rétrospective descriptive allant du janvier 2006 à décembre 2010, réalisée dans le service de Médecine Interne de l'hôpital de Pikine de Dakar. Etaient inclus, tous les malades ayant subi une biopsie hépatique, en ambulatoire ou en hospitalisation. Etaient exclus, tous les patients qui ont bénéficié d'une PBH dont les résultats n'ont pas été retrouvés. Les données suivantes étaient étudié: l’âge, le sexe, les indications, la taille du fragment biopsique, Le nombre de carottes, les complications, la comparaison des diagnostics pré biopsiques avec les comptes rendus histologiques.

**Résultats:**

Ont été colligées 70 patients atteints d'hépatopathie chronique ayant bénéficiés d'une PBH. Il s'agissait de 46 hommes (65.71%) et 24 femmes (34.29%). L’âge moyen des patients était de 36 ans. Les PBH étaient réalisées en ambulatoire chez 58 patients (82.86%) et chez 12 malades hospitalisés (17,14%). Les indications étaient dominées par les hépatites virales chroniques dans 62,86% (44cas), suivi des processus tumoraux du foie dans 24.29% (17 cas). Les complications survenues chez 15 patients (21.43%) étaient représentées de 14 cas de douleur (20%) et d'un cas de malaise vagal (1.43%). Les 70 PBH effectuées ont ramené une carotte dans 35,71% des cas, 2 à 6 carottes dans 32,87% des cas. La longueur moyenne des fragments biopsiques était de 22 ±8 mm. Soixante-six résultats étaient interprétables et 4 non interprétables soit une performance diagnostique de 94,29%.

**Conclusion:**

La PBH est de pratique sure, avec un respect des contres indications et une bonne maitrise de la technique. Son acceptabilité a été bonne dans notre pratique et sa rentabilité diagnostique excellente. Elle devrait être beaucoup plus vulgarisée dans notre pays, comme alternative aux moyens d'exploration non invasive.

## Introduction

La ponction biopsie hépatique demeure une des alternatives pour l'exploration précise et l’évaluation des hépatopathies chroniques dans les pays à moyens limités. Elle est indiquée, pour l’établissement d'un diagnostic, d'un pronostic et d'un traitement qui sont presque toujours fonction des résultats anatomopathologiques [1]. Des tests non invasifs ont été validés en Occident pour avoir de moins en moins recours à la PBH pour l’évaluation d'une fibrose hépatique [2, 3]. Une seule série portant sur les PBH a été rapportée au Sénégal [4]. Les objectifs de notre travail ont été de: déterminer ses indications couplée à l’étude anatomo-patholgique des prélèvements, déterminer les incidents et accidents liés à cette technique invasive, formuler des recommandations concernant la prise en charge des hépatopathies chroniques dans les pays à moyens limités.

## Méthodes

Il s'agissait d'une étude rétrospective descriptive allant de janvier 2006 à décembre 2010, réalisée dans le service de Médecine Interne du CHN de Pikine, de la région de Dakar. Etaient inclus, tous les malades atteints d'une hépatopathie chronique ayant bénéficié d'une biopsie hépatique suivis soit en ambulatoire (référés ou non), soit en hospitalisation. Etaient exclus, tous les patients atteints d'hépatopathies chroniques dont les dossiers étaient inexploitables. Les PBH ont été réalisées par 2 médecins du service de médecine interne. L'aiguille de type aspiratif (Hepafix* 16 G/1,6mm, Braun MedicalMelsungen, Germany) a été le seul type d'aiguille utilisé chez tous les patients.

Le consentement oral du malade était recueilli après explication de la procédure. Un bilan de la crase sanguine satisfaisant était exigé: taux de prothrombine et de plaquettes respectivement supérieurs à 60% et à 100 000 plaquettes / mm3. Toutes les biopsies étaient écho guidées. En cas de ponction blanche, une deuxième tentative était réalisée. Une surveillance de l′état hémodynamique du patient (pouls, tension artérielle), et un examen de l'abdomen étaient régulièrement effectués toutes les 30 minutes pendant 4 heures. Un traitement à base de paracétamol ou de nefopam était administré en cas de douleur.

Les paramètres suivants ont été analysés: l’âge, le sexe, les indications, la technique de la PBH, la taille du fragment et le nombre de carotte prélevé. Ces prélèvements étaient mis dans un flacon contenant du formol puis acheminés dans la journée au laboratoire d'anatomopathologie. En ce qui concerne les incidents ou accidents post biopsiques, étaient recherchés: une douleur, un malaise vagal, une hémorragique, une ponction d'organe de voisinage. Les résultats histologiques ont été classés en pathologies néoplasiques primitives ou secondaires et non néoplasiques. Les cirrhoses et hépatites chroniques virales répertoriées selon le score de Métavir et des autres hépatopathies chroniques. L'apport de la PBH au diagnostic des hépatopathies était fait en comparant les diagnostics pré biopsiques avec les comptes rendus histologiques.

## Résultats

Dans une période de 6 ans, 70 cas de PBH étaient colligées. Il s'agissait de 46 hommes (65.71%) et 24 femmes (34.29%), soit un sex-ratio de 1.9. L’âge moyen des patients était de 36 ans avec des âges extrêmes de 2 et 75 ans. Les PBH étaient réalisées en ambulatoire dans 82.86% des cas ce qui représentait 58 patients de la population d’étude.

**Diagnostics pré biopsiques:** Les indications étaient dominées par l’évaluation d'une hépatite virale chronique. Six cas n'appartenaient à aucune des entités anatomo-clinques sus-citées; il s'agissait d'un cas suspect de fibrose congénitale, d'un cas suspect de granulomatose, d'un cas de cytolyse hépatique récidivante, d'un cas de syndrome d'hypertension portale et deux cas de suspicion d'hépatite auto-immune. Pour 3 cas de PBF, l'indication n'avait pas été précisée (Tableau 1).

**Tableau 1:** Indications des ponction biopsie hépatique

Type d'hépatopathie	Nombre de cas	Pourcentage
Hépatite virale chronique	44	62.86
Hépatite B	41	58.57
Hépatite C	2	2.86
Hépatite B + C	1	1.43
Foie tumoral	17	25.28
Suspicion d'hépatite auto-immune	2	2.86
Autres	4	5.72
Indication non précisée	3	4.28
**Total**	**70**	**100**

**Complications:** Des complications étaient survenues chez 15 patients, soit 21.43%. Elles étaient représentées de 14 cas de douleur soit 20% et d'un cas de malaise vagal soit 1.43%. Aucun cas d'hémorragie ou de ponction accidentel d'un organe voisin n'a été observé.

**Prélèvements:** Les 70 PBH effectuées ont ramené une carotte dans 35.71% des cas, 2 à 6 carottes dans 32.87% des cas, chez 18 patients (25.71%) le prélèvement était très fragmenté, chez 4 autres le nombre de carottes n’était pas précisé (5,71%). La longueur moyenne des fragments biopsiques était de 22 ± 8 mm avec des valeurs extrêmes allant de 4 mm à 36 mm.

**Diagnostics histologiques:** Sur les 70 PBH réalisées, 66 résultats étaient interprétables et 4 non interprétables soit un rendement diagnostique de 94.29%. Pour les 4 biopsies non interprétables (5.71%), il s'agissait de prélèvements insuffisants avec de petits fragments d'une taille moyenne de 4mm comptant moins de 2 espaces portes. Le Tableau 2 affiche les différentes conclusions des examens anatomopathologiques interprétables. Pour les hépatites virales chroniques, il s'agissait de 41 cas d'hépatite B, 1 cas d'hépatite C, 1 cas d'hépatite B + C. Les 7 cas de localisations tumorales secondaires du foie comprenait: 5 cas d'adénocarcinomes dont 1 adénocarcinome peu différencié à cellules claires d'origine non précisée, 2 adénocarcinomes d'origine colique respectivement peu différencié et bien différencié, 1 adénocarcinome bien différencié d'origine excréto-bilio-pancréatique et 1 adénocarcinome bien différencié d'origine non précisée. Pour 2 cas de localisations tumorales secondaires, la nature était non précisée. Les 6 cas de de cancers primitifs se répartissaient en 4 cas de carcinome hépatocellulaire, 1 cas de cholangiocarcinome et 1cas d'hépato-cholangiocarcinome. Les 4 cas de cirrhoses étaient post-hépatitique B dans 2 cas, post-hépatitique C dans 1 cas et d’étiologie non précisée dans 1 cas. Les 6 résultats classés « autres » correspondaient à 3 résultats histologiques normaux, 1 cas de stéatose macro vacuolaire massive isolée, 1 cas de veinopathie portale oblitérante sur lupus systémique, 1 cas de tumeur bénigne à cellules fusiformes.

**Tableau 2:** Diagnostics anatomo-pathologiques

Diagnostics	Nombre de cas	Pourcentage
Hépatites virales chroniques	43	61.42
Localisations tumorales secondaires	7	10
Cancers primitifs	6	8.59
Cirrhoses	4	5.70
Autres	6	8.59
Résultats sans diagnostic	4	5.70
**Total**	**70**	**100**

**Discordance entre diagnostics pré biopsiques et résultats histologiques:** Parmi les 66 biopsies interprétables, l'histologie avait permis de redresser la suspicion diagnostique dans 5 cas soit 7.57%. Il s'agissait: d'un examen normal respectivement pour 1 cas suspect de fibrose hépatique congénitale, 1 tableau clinique de cytolyse hépatique récidivante. Pour un malade chez lequel était suspecté une granulomatose, la PBH a conclu une stéatose macro vacuolaire massive isolée. Un carcinome hépatocellulaire a été retrouvé dans 1 cas de suspicion de localisation secondaire hépatique. Une cirrhose était retrouvée chez un malade porteur d'une hépatomégalie multi nodulaire d'allure tumorale.

## Discussion

**Indications:** L′analyse histologique d'un fragment du foie obtenu par ponction biopsie hépatique reste aujourd'hui un élément essentiel pour le diagnostic de nombreuses maladies chroniques du foie. Dans pays nantis, les indications de la PBH se sont considérablement modifiées au cours de ces dernières années du fait du développement de tests non invasifs sensibles et spécifiques pour le diagnostic de plusieurs maladies chroniques du foie [2].

Dans notre étude, les indications étaient dominées par l’évaluation d'une hépatite virale B chronique dans 58.57%. Dans celle de Sombié et al en 2009 au Burkina Faso [5], les hépatites chroniques B en représentaient 75.4%. Ces valeurs témoignent de la forte prévalence de l'hépatite B en Afrique de l'Ouest surtout parmi la population active, comme en témoigne l’âge jeune de nos patients (36 ans). Les indications étaient dominées par les cancers primitifs du foie dans 42.7%, alors que les hépatites chroniques ne représentaient que 1,8% dans le travail de Mbengue et al en 2003 à Dakar [4]. La disponibilité du Ténofovir, pour le traitement des hépatites chroniques B, explique en partie la modification du profil des indications à Dakar. Dans les pays nantis, les indications sont dominées par l'hépatite chronique C avec des comorbidités, l'hépatite chronique B, suivies des tumeurs hépatiques, des hépatopathies d'origine métabolique et de la transplantation hépatique [1, 6, 7]. La PBH était effectuée pour la quasi-totalité de nos patients (82.86%) en ambulatoire. Sa réalisation en hôpital de jour ne présente pas de risques supplémentaires par rapport aux malades hospitalisés. Plusieurs équipes françaises ont montré que la PBH ambulatoire était un procédé sûr et efficace qui diminuait l'inconfort du malade et augmentait l'acceptabilité d'un examen ultérieur réalisé dans les mêmes conditions tout en réduisant son coût financier [1, 8].

Aucune prémédication n'a été réalisée chez nos patients. Cependant, certains auteurs préconisent une sédation intraveineuse [9]; ce qui nous semble pertinent pour des raisons d’éthique médicale. Un repérage échographique a été réalisé chez tous nos patients. Selon Younossi et al [10], le risque de survenue d'incidents et/ou d'accidents post BPH était 2 fois plus fréquent lors des PBH sans repérage échographique. Plusieurs auteurs [11–13] ont montré que les complications hémorragiques et les perforations d'organes étaient moins fréquentes après repérage échographique comparé aux biopsies faites à l'aveuglette. Le guidage échographique qui du reste est toujours indiqué, permet un meilleur repérage du site de ponction pour les lésions focales [14, 15].

La longueur moyenne des fragments biopsiques dans notre travail, était de 22 ± 8 mm. Il est habituellement admis que 25 mm est une longueur assurant une bonne fiabilité des résultats de l'examen histopathologique, notamment dans l’évaluation du stade de fibrose [16]. Une longueur de 15 mm est la longueur minimale acceptable, même si elle entraîne déjà un degré d'incertitude relativement élevé dans l’évaluation de certains paramètres histopathologique, dont le stade de fibrose [17].

**Incidentes et/ou accidents:** Dans notre travail, les incidents de la PBH étaient représentés par une douleur dans 21.43%. Selon Gilmore et al [18], la douleur après PBH se situait entre 20 et 30%. Elle survenait dans 60.5% et le malaise vagal dans 3,9% dans l’étude de Sombié et al [5], La différence avec cette dernière pourrait s'expliquer par l'utilisation d'aiguille de gros diamètre 14 G et 15 G. Le diamètre plus petit de l'aiguille a une influence favorable sur les manifestations douloureuses. P

armi les incidents mineurs, un malaise vagal survient dans 0.4 à 3% des PBH [19]. La fréquence du malaise vagal dans notre étude de 1.43% est superposable à celle de la littérature. Aucun cas d'accident n'a été retrouvé dans notre étude. En principe, avec un bon repérage échographique, on peut éviter l'atteinte de la sphère pancréato-vésiculaire. Mbengue et al [4] avaient obtenu 2 cas d'hémorragies avec hémopéritoine et un cas d'ensemencement pariétal. Dans d'autres séries [12, 20, 21], le risque d'accidents post PBH était évalué entre 0.09 et 2.35%. L'aiguille de 16 ou 18 G semble être plus adaptée, permettant à la fois l'obtention d'un bon fragment avec moins d'accidents hémorragiques [16, 21, 22]. Les paramètres d'hémostase pré requis pour réaliser une PBH transpariétale sont assez différents d'une étude à l'autre, sans aucun niveau de preuve [15]. Un taux de prothrombine supérieur 50% et un taux de plaquettes supérieur 70000/mm3, paraissent acceptables à la réalisation d'une PBH transpariétale [1]. Lorsque ces normes sont respectées, le risque hémorragique est plus faible.

**Valeur diagnostique de la PBH:** Dans notre étude le rendement diagnostique de l'histologie était de 94.29%. Spycher et al [23] sur un travail portant sur 411 PBH rapportaient un rendement diagnostique de 84.4%; avec une discordance entre le diagnostic pré biopsique et le compte rendu histologique final dans 6.8% (28 cas). L'examen histopathologique du prélèvement est soumis aux biais et aux aléas de l’échantillonnage. Ainsi le contact régulier entre le clinicien et le pathologiste est important pour garantir une qualité constante du prélèvement. D'une façon générale, il existe très peu d'informations sur l’évaluation de la qualité diagnostique proprement dite de la PBH et surtout sur la fréquence des erreurs diagnostiques [16].

## Conclusion

La place de la PBH dans la prise en charge d'un patient atteint d'une hépatopathie chronique a beaucoup évolué ces dernières années surtout dans les pays nantis. Avec la forte prévalence de l'hépatite chronique virale B dans nos régions où les tests non invasifs d’évaluation de la fibrose font défaut pour le moment du fait de leur inaccessibilité financière vis-à-vis de nos populations; la PBH demeure encore incontournable dans le diagnostic et l’évaluation de l'histoire naturelle des hépatopathies chroniques. Sa pratique en ambulatoire, avec un respect strict des contre-indications et un bon repérage échographique est de pratique sure. Les complications sévères peuvent être nulles. L'obtention d'un rendement histologique efficient passe par le recueil de carottes significatives.
